# Perinatal depressive symptoms among low-income South African women at risk of depression: trajectories and predictors

**DOI:** 10.1186/s12884-019-2355-y

**Published:** 2019-06-14

**Authors:** Emily C. Garman, Marguerite Schneider, Crick Lund

**Affiliations:** 1 Alan J Flisher Centre for Public Mental Health, Department of Psychiatry and Mental Health, University of Cape Town, Cape Town, South Africa; 2 Centre for Global Mental Health, Health Service and Population Research Department, Institute of Psychiatry, Psychology and Neuroscience, King’s College London, London, UK

**Keywords:** Trajectory, Depression, Perinatal, Risk factors, Low-income

## Abstract

**Background:**

The aim of the study was to identify trajectories of perinatal depressive symptoms and their predictors among women living in a low-resource setting in South Africa, and who present with a risk of depression during pregnancy.

**Methods:**

This is a secondary analysis of a randomised controlled trial among 384 women living in Khayelitsha, a low income setting in South Africa, recruited at their first antenatal visit if they scored 13 or above on the Edinburgh Postnatal Depression Scale, were at least 18 years of age, less than 29 weeks pregnant and spoke isiXhosa. Participants were followed up at 8 months gestation, 3 and 12 months postpartum. Latent trajectories of depressive symptoms were identified using growth mixture modelling, based on the Hamilton Depression Rating Scale (HDRS). There were no differences in HDRS scores between the control and intervention arms, so all participants were assessed together. Health, social and economic predictors of trajectories were investigated to identify high-risk groups with greater or more chronic depressive symptoms, using univariate logistic regression.

**Results:**

Two trajectories were identified: *antenatal only* (91.4%), with moderate to severe symptoms at baseline which later subside; and *antenatal and postnatal* (8.6%), with severe depressive symptoms during pregnancy and later in the postpartum period, which subside temporarily to moderate levels at 3 months postpartum. Predictors for the *antenatal and postnatal* trajectory include severe food insecurity, intimate partner violence, lower social support, greater functional impairment, problematic drinking and suicide risk.

**Conclusions:**

A small proportion of women who are at risk for depression antenatally remain at risk throughout the perinatal period, and can be differentiated from those who show a natural remission. Identification and referral strategies should be developed with these findings in mind, especially given the limited mental health resources in low-income settings.

**Electronic supplementary material:**

The online version of this article (10.1186/s12884-019-2355-y) contains supplementary material, which is available to authorized users.

## Background

Depression during pregnancy and the postnatal period, known as perinatal depression, is a concern worldwide. In South Africa, the prevalence of women at high risk of depression or suffering from depression ranges between 21 and 39% antenatally [1–4], and between 16 and 32% postnatally [5–7]. The burden of disease associated with perinatal depression and impact on child health and development [8, 9] warrants further research to understand the disorder’s aetiology, identify at-risk populations and develop effective preventive and therapeutic interventions.

The global evidence base on risk factors for perinatal depression is growing. Several factors have systematically been reported in low- and middle-income countries (LMICs) and high-income countries (HICs), such as a history of depression, social conflict, and lack of social support from family or partner [10–13]. Younger age, lower education status and being single are among the few demographic risk factors which have received some, but mixed evidence, for both antenatal and postnatal depression [9, 11–13]. The evidence for socio-economic risk factors is mixed in South Africa [3, 4, 14]. However, food insecurity, defined as the inability to access a sufficient quantity of healthy food on a daily basis and reported by 38% of households in South Africa [15], has consistently been identified as a risk factor for antenatal and postnatal depression in the Western Cape [5, 14, 16, 17]. Intimate partner violence (IPV) has also been reported as a risk factor for perinatal depression in South Africa [3, 18–20], where IPV is common and is reported by more than 40% of pregnant women [21].

Fewer studies have focused on identifying health-related predictors of perinatal depression, yet these are particularly relevant in LMICs. In South Africa, the prevalence rate of women aged 15 to 49 living with HIV is approximately 24% [22], and evidence indicates that perinatal depression is more common among HIV-positive women [19]. The rates of alcohol consumption per capita are also very high in South Africa [23]: in recent studies conducted in low-income areas of Cape Town, hazardous drinking was reported by 7.1% of women at 8 months gestation [24], and by 16% of women three months after giving birth, indicating a level of alcohol consumption likely to have adverse health consequences [5]. Evidence suggests an association between alcohol use during pregnancy and postpartum depression [5, 24], though none has been found with antenatal depression [1].

The heterogeneity in risk factors identified for antenatal or postnatal depression highlight the complexity of this disorder’s aetiology and course. The fact that most of the evidence is based on cross-sectional studies further limits our understanding of the factors associated with the onset, severity and chronicity of depressive symptoms during the perinatal period. Recent literature has used latent modelling techniques to investigate the heterogeneity of depression, both in terms of symptom profiles and trajectories [25]. Two systematic reviews have summarised the evidence using such modelling techniques in the context of perinatal depressive symptoms [26, 27]. Both reviews identified the most commonly reported trajectories to be a chronically severe and a chronically low symptom level trajectory. Transient trajectories were also reported, some of which suggested a natural remission among some women, despite similar severe levels of depressive symptoms antenatally compared to those suffering from chronic severe symptoms throughout the perinatal period [28–31]. Baron et al. [26] also point that predictors identified for several trajectories were not consistent across studies and did not distinguish women with chronic symptoms from those who presented transient trajectories.

Identifying such predictors would be especially useful in low-resource settings such as South Africa, since the use of screening instruments to identify women at risk of depression, without effective referral and treatment mechanisms, can overburden already weak and limited mental health services [32]. Indeed, being able to identify women who are most likely to suffer from chronic symptoms from those whose symptoms may abate naturally with minimal intervention may help streamline referrals and help target women who are most at risk. Unfortunately, as both reviews highlight, there is a dearth of evidence from LMICs. Only one LMIC study was conducted, among West African perinatal women [33]. The inclusion criteria meant, however, that the sample was a particularly low-risk group, and neither chronically severe or initially severe trajectories were identified. Given the gap in the literature, the aim of this study was to identify trajectories of perinatal depressive symptoms and their predictors among low-income South African women who were already at risk of depression during pregnancy.

## Methods

### Design and setting

This study is a secondary analysis of data collected for a randomised controlled trial (RCT) assessing the cost-effectiveness of a brief psychosocial intervention for perinatal depression among 425 pregnant women at risk of depression living in Khayelitsha, a peri-urban informal settlement on the outskirts of Cape Town, South Africa. The poor living conditions, high crime rates and population density of Khayelitsha resembles that of the other informal settlements in South Africa [34–36]. The psychosocial intervention did not have an effect on women’s depressive symptoms [37], which allowed the use of this sample for the purpose of the present study. The recruitment and data collection methods have been described previously [38], and are briefly reviewed here.

### Participants

Recruitment took place in two community health centres in Khayelitsha. Pregnant women were screened for depressive symptoms during their first antenatal clinic booking, using the Edinburgh Postnatal Depression Scale (EPDS; [39]). The EPDS is a 10-item Likert-scale questionnaire assessing a range of depressive symptoms, such as anhedonia, somatic symptoms and suicide ideation. Its internal structure was acceptable among isiXhosa-speaking women in Khayelitsha [40]. Another validation study, conducted in an informal settlement in Johannesburg, suggests that a cut-off of 13 is optimal to indicate a risk for depression, with a sensitivity and specificity of 80 and 76.6%, respectively [41]. Women who were at least 18 years of age, spoke isiXhosa, were in their first or second trimester and scored 13 or above on the EPDS were eligible for enrolment. For this study, participants of babies who had low birth weight (< 2.5 kg; *n* = 27) or who were premature (< 37 weeks gestation; *n* = 43) were included in the analysis. However, participants who died, who experienced a miscarriage, or whose baby died during the course of the study were excluded from the analyses (*n* = 42). Besides greater levels of functioning among participants excluded from the analysis (median = 19.4; interquartile range (IQR) = 8.3–30.6) compared to those included (median = 29.2; IQR = 16.7–41.7; U = -2.64; *p* = 0.008)-, the baseline demographic, clinical or social characteristics of participants excluded from and included in the analysis did not differ [37].

### Procedure

Once enrolled, participants were randomised into either a psychosocial intervention or enhanced usual care. The psychosocial intervention was provided by trained community health workers and consisted of six counselling sessions which included elements of psycho-education on depression and pregnancy, problem solving, behavioural activation and healthy thinking [42]. The enhanced usual care consisted of monthly phone calls for three months, where participants were asked a series of question relating to their health, suicide risk and recent life events. Phone calls lasted no more than five minutes, and were conducted by two separate community health workers, who were trained to conduct the phone calls, but were not trained in counselling. More details about the interventions and training are provided in Lund et al. [38]. All participants received the same regular antenatal care available at the clinics, which typically involves medical management of pregnancy, HIV testing and Prevention of Mother to Child Transmission care. An assessment was conducted at recruitment, and then again at eight months gestation, and three months and 12 months after giving birth. This was done by two fieldworkers who were blind to the participant’s arm allocation.

### Measurements

All assessments covered a range of mental health, health, social and economic measures. The baseline assessment also included socio-demographic questions [38]. Only age, education and marital status were considered potential demographic predictors and included in the analyses, as these characteristics are routinely collected during the first antenatal visits in South Africa.

### Health characteristics

Depressive symptoms were primarily assessed using Potts et al. [43]‘s 17-item version of the Hamilton Depression Rating Scale (HDRS) [44]. Scores range from 0 to 54; a higher score suggesting greater symptom severity. A cut-off of 17 has been suggested as indicating clinically significant depressive symptoms [45]. A more structured isiXhosa version of the HDRS was developed for the RCT for use by non-clinicians: this adapted version was validated and showed good construct validity and internal consistency (Cronbach’s Alpha = 0.74), and the inter-rater (0.97 to 0.98) and test-retest reliability (0.90) were excellent [46].

The Mini International Neuropsychiatric Interview (MINI) 6.0 [47] Major Depressive Episode and Suicidality modules were used to assess current depression and suicidality risk, respectively. A lifetime diagnosis of depression was also assessed using the Major Depressive Episode module. The MINI 6.0 is a brief diagnostic interview which has been used as a gold standard in diverse populations, including among HIV-infected patients in South Africa [48, 49]. High risk of suicide was defined as a score of 17 or more [50]. Participants who reported a high risk of suicide were immediately referred to see a psychiatric nurse, located in the same community health centre.

The World Health Organization (WHO) Disability Assessment Schedule (WHODAS 2.0; 12-item) [51] was used to determine the participants’ level of impaired functioning. The item-response theory-based scoring was used, generating a score between 0 and 100, with greater scores suggesting greater impairment. The WHODAS 2.0 has good reliability and validity across cultures and population type [51]. As recommended by Schneider et al. [52], the WHODAS 2.0 was complemented with the Cape Town Functional Assessment Instrument (FAI), developed specifically for and validated among pregnant and postnatal women in this study and is meant to reflect more specific domains of functioning among this population [52]. It is a 10-item questionnaire, with responses ranging from “no difficulty” to “can never do the task”. A “not applicable” option is also available, so total scores are calculated by dividing the sum of item scores by the number of items responded to. The total score ranges from 0 to 4, with a greater score suggesting greater impairment.

Finally, alcohol use was assessed using the Alcohol Use Disorder Identification Test (AUDIT) [53], a 10-item questionnaire, developed by the WHO, to identify alcohol misuse. Scores range from 0 to 40, with greater scores indicating greater alcohol misuse. The AUDIT has been used to assess alcohol consumption habits in both men and women in the Cape Town region [54, 55]. The recommended cut-off for heavy or binge drinking among women in South Africa is 5, based on a validation study among a nationally representative sample [56]. HIV status was also recorded.

### Social characteristics

The Multidimensional Scale of Perceived Social Support (MSPSS) [57], a 12-item 7-point Likert Scale questionnaire, was used to assess perceived emotional support from family, friends and a ‘special person’. Here, a ‘special person’ refers to a significant other, or a person with whom the participant has a close emotional relationship and is involved in their day-to-day lives. Overall scores range from 0 to 84, with higher scores suggesting greater perceived support. The scale has been validated in several LMICs [58, 59], including high-school students in Cape Town, South Africa [60, 61]. Subscale scores were also calculated. IPV was assessed by asking participants if they had experienced physical (e.g. kicked, slapped, beaten) or sexual abuse by their partner in the past three months.

### Economic characteristics

The Household Food Insecurity Access Scale (HFIAS) [62] is a 9-item questionnaire assessing three dimensions of food insecurity. It was previously used in a study among postnatal depressed women in Khayelitsha [5]. The HFIAS score was binarised so that participants were either considered severely food insecure or not, as done in previous research in other low-income settings [5, 63]. A proxy for socio-economic status was also developed using multiple correspondence analysis, where economic-related variables were analysed to create an asset-based score [64]. Variables included, but were not limited to, education, employment status, main source of income, whether the household income is fixed, housing characteristics and access to amenities. The score was transformed into a binary variable indicating whether participants were in the lower (below median asset score) or higher wealth category (at or above median asset score).

Instruments which had already been translated and validated in isiXhosa in previous studies (such as the EPDS, MINI, AUDIT, FAI and HFIAS) were reviewed by a translator for accuracy. All other sections in the assessments were translated into isiXhosa and back-translated to English.

### Analysis

#### Identification of trajectories

The first stage of the analysis, conducted in Mplus version 8 [65], consisted of conducting growth mixture modelling (GMM), a method which combines growth curves with latent modelling. GMM allows investigators to explore groups of individuals with similar profile trajectories (classes) and allows for individual variability within latent classes. The HDRS scores at the four timepoints (recruitment, 8 months gestation, and 3 and 12 months postpartum) were used to create latent trajectories. This instrument, rather than the EPDS, was used as it more sensitive to change [66].

Scores on the HDRS did not differ significantly between the control and intervention arms at any assessment [37], so participants from the two arms were analysed together. However, attrition in the intervention arm (19.6%) was higher than that in the control arm (6.5%), though no differences were found in baseline characteristics between participants who were lost to follow-up and those who were followed-up. To account for differences in attrition, arm allocation was included as a covariate in all growth mixture models. Missing data were assumed to be missing at random and were dealt with using robust maximum likelihood estimation. To represent non-equidistant time points of assessments, factor loadings were fixed to 0, 0.3, 0.7 and 1.6, to represent assessments at baseline, and then 3 months, 7 months and 16 months after baseline; time in months was divided by 10 to avoid non-convergence of the models [67]. An inspection of the individual data suggested heterogeneous, non-linear trends. Goodness of fit values generated from preliminary one-class (non-mixture) analyses indicated that a quadratic change function fitted the data best [68], so a quadratic pattern was introduced in subsequent models (see Additional file 1).

A series of mixture models were run, first assuming no variation within trajectory classes (intercepts and slope variance fixed at 0 – latent class growth analysis [LCGA]), and then allowing free estimates of means and variances for latent variables (GMM). Small and non-significant negative residual variances for HDRS scores at 12 months were dealt with by fixing the residual variance to a value close to 0 [67].

#### Selection of optimal model

Models with increasing number of classes were fitted against the data, and compared using standard statistical measures: the Bayesian Information Criterion (BIC) [69], the Akaike Information Criterion (AIC) [70] and entropy [71]. Priority was given to entropy in cases where fit indices between two models were relatively similar [72]. Given the relatively small sample size of the RCT, model solutions that included a class that comprised less than 5% of the sample were avoided. Average probability of class membership for each estimated class (posterior probability) were also used as a criterion for model fit. Successive models with different number of classes were compared using the Lo-Mendell-Rubin Test (LMRT) [73] and Bootstrap Likelihood Ratio Test (BLRT) [74]. Finally, the shape and theoretical interpretability of the trajectory classes were also taken into account. Once the optimal model was selected, participants were assigned to a latent trajectory class based on their highest posterior probability.

To assess whether the psychosocial intervention had an effect on latent trajectories generated by the GMM, a sensitivity analysis was also conducted by running an unadjusted GMM, this time including the arm variable within the GMM model, so that trajectories would be generated per arm [75, 76].

#### Identification of risk factors

In the second stage of the analysis conducted in Stata 14, each demographic, health, social and economic variable collected at baseline was entered as a single predictor in an unadjusted logistic regression, with class membership as the outcome. Odds ratios (OR) with 95% confidence intervals are reported, with a significance level set at 5%. Univariate, rather than multivariate, analyses were preferable given that the objective of the study was to identify high-risk groups more likely to suffer from severe and chronic symptoms, rather than to understand the complex interactions of risk factors leading to chronic depressive symptoms.

## Results

### Overview of sample

The characteristics of the sample at baseline are presented in Table 1. On average, participants were 27 years of age (standard deviation (SD) = 5.56). The majority of participants reported not finishing high school (*n* = 225, 58.6%), not living with a partner (*n* = 254, 66.2%), and were not employed or still studying (*n* = 207, 53.9%). Over a quarter reported being severely food insecure (*n* = 112, 29.2%). A similar proportion reported being HIV-positive (n = 112, 30.1%) and drinking heavily during pregnancy (*n* = 114, 29.7%). Over 40% (*n* = 157) were diagnosed with current depression on the MINI, 17.7% (*n* = 68) were considered at risk for suicide, and 31.8% (*n* = 122) had a history of depression. The mean number of assessments conducted was 3.5; 88.0% (*n* = 338) of participants received at least three assessments, and 65.6% (*n* = 252) received all four assessments.

**Table 1:** Characteristics of the sample, by class

Variable	Total (*N* = 384)			Antenatal only (*N* = 351)			Antenatal & postnatal (*N* = 33)		
	n	%	Mean (SD)	n	%	Mean (SD)	n	%	Mean (SD)
Demographic characteristics									
Age			27.2 (5.61)			27.0 (5.54)			28.7 (6.23)
Gestation			17.2 (5.71)			17.1 (5.74)			18.2 (5.31)
Did not complete high school	225	58.6		203	57.8		22	66.7	
VDoesn’t live with partner	254	66.2		232	66.1		22	66.7	
Economic measures									
Unemployed/studying	207	53.9		190	54.1		17	51.5	
Lower wealth (socio-economic status)	195	50.8		176	50.1		19	57.6	
Severely food insecure	112	29.2		96	27.4		16	48.5	
Social characteristics									
Intimate partner violence (past 3 months)	50	13.0		41	11.7		9	27.3	
Overall social support			58.3 (12.91)			58.8 (12.56)			52.8 (15.3)
Social support from family			20.1 (5.70)			20.4 (5.48)			16.9 (6.97)
Social support from friends			16.0 (6.24)			16.0 (6.19)			15.3 (6.77)
Social support from special person			22.2 (4.75)			22.4 (4.57)			20.6 (6.18)
Health characteristics									
Functioning (WHODAS)			29.8 (17.64)			28.9 (17.13)			39.3 (20.34)
Functioning (FAI)			0.8 (0.5)			0.8 (0.5)			1.2 (0.6)
Heavy drinking	114	29.7		99	28.2		15	45.5	
HIV positive status	112	30.1		100	29.3		12	38.7	
Current diagnosis of depression (MINI)	157	40.9		136	38.8		21	63.6	
Lifetime diagnosis of depression (MINI)	122	31.8		104	29.6		18	54.6	
High suicide risk (MINI)	68	17.7		57	16.2		11	33.3	

*SD* = standard deviation

### Identification of trajectories

Table 2 provides the fit information criteria of the models generated through LCGA and GMM. Smaller values of BIC and AIC suggest a better model fit, while entropy values closest to 1 suggest better classification. Models generated through GMM fitted the data better than the LCGA models. Fit indices indicated that a 2-class model was optimal (BIC = 7797.269; AIC = 7722.207), with an entropy (0.816) above the suggested minimum of 0.8 [77]. The 3-class model had a slightly lower AIC value (7708.730) and generated a small but interesting class characterised by a clinically different trend compared to the other two classes (see Additional file 2 for a graphical representation of the trajectories). However, the entropy was lower, and the model fit not significantly improved from the 2-class model according to the LMRT and BLRT. Taking these into consideration, and for reasons of parsimony, the 2-class model was selected.

**Table 2:** Latent class growth analysis and growth mixture modelling: comparisons of models

Classes	BIC	AIC	Entropy	Size (%) of smallest class	LMRT statistic (*p*-value)	BLRT statistic (*p*-value)
Quadratic LCGA^a^						
2	7825.996	7770.687	0.742	26.0	203.838 (< 0.001)	− 3977.544 (< 0.001)
3	7809.547	7738.435	0.750	4.6	38.630 (0.034)	− 3871.344 (0.030)
4	7817.215	7730.301	0.718	4.6	15.483 (0.304)	− 3851.218 (0.288)
5	7828.413	7725.696	0.584	3.9	12.097 (0.657)	− 3843.151 (0.647)
Quadratic GMM ^b^						
**2**	**7797.269**	**7722.207**	**0.816**	**8.6**	**38.789 (< 0.001)**	**− 3862.313 (< 0.001)**
3	7799.594	7708.730	0.807	4.2	20.612 (0.503)	− 3842.104 (0.488)
4	7814.876	7708.209	0.762	1.6	8.177 (0.234)	− 3831.365 (0.229)
5	7822.705	7700.235	0.771	1.3	15.330 (0.123)	− 3827.105 (0.115)

*AIC* = Akaike Information Criterion, *BIC*=Bayesian Information Criterion; *BLRT* = Bootstrap Likelihood Ratio Test, *LMRT* = Lo-Mendell-Rubin Test; ^a^Latent curve growth analysis; ^b^growth mixture modelling

Note: the final model selected is indicated in bold

**Fig. 1:**
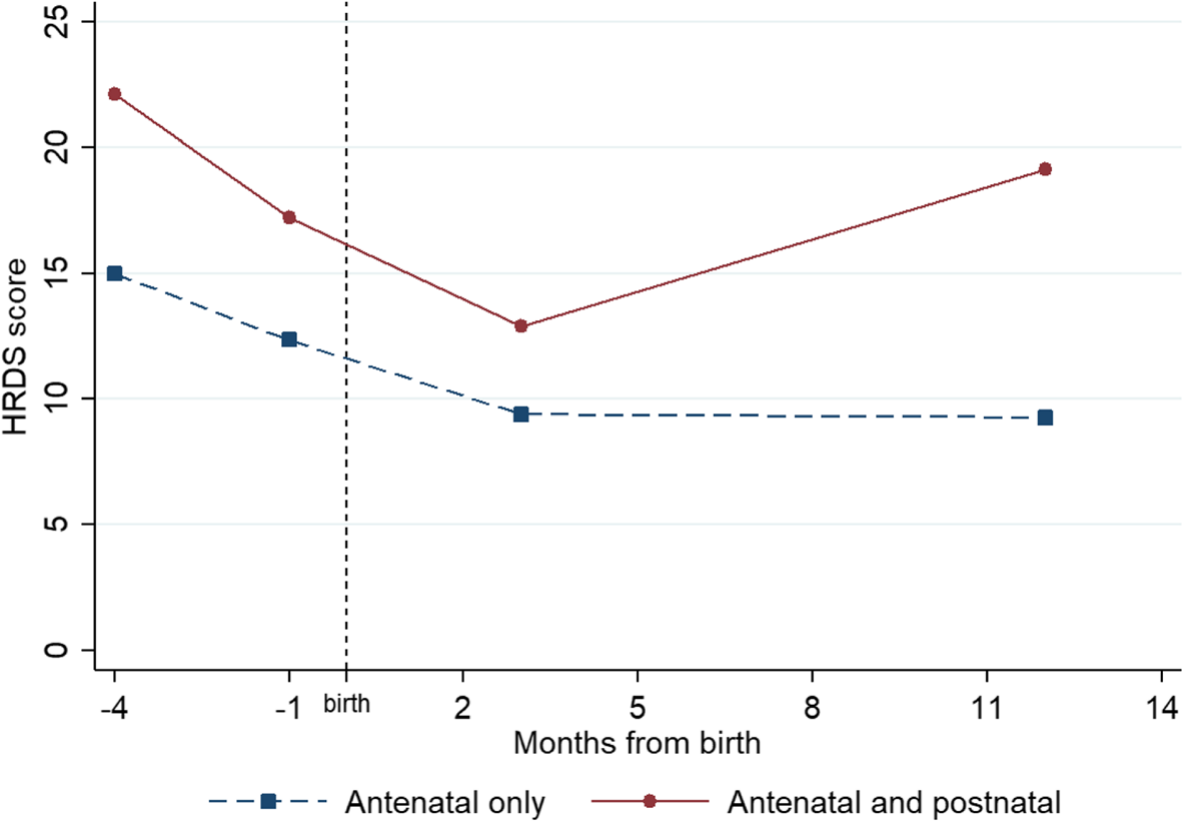
Mean HDRS curves of the GMM 2-class solution; legend: Note: Missing data for *antenatal only* trajectory: 8 months gestation (*n* = 84, 23.9%), 3 months postpartum (*n* = 43, 12.3%) and 12 months postpartum (*n* = 61, 17.4%); missing data for *antenatal and postnatal* trajectory: 8 months gestation (*n* = 10, 30.3%), 3 months postpartum (n = 4, 12.1%) and 12 months postpartum (*n* = 3, 9.1%)

The mean HDRS scores of women allocated to the two classes are presented in Fig. 1. The *antenatal only* class is characterised by moderate levels of depressive symptoms at recruitment (mean = 15.0, SD = 4.28), which decrease steadily over pregnancy and early postpartum, then stabilise by 12 months postpartum (mean = 9.3, SD = 3.82). The majority of the sample were allocated to this trajectory (*n* = 351, 91.4%). The *antenatal and postnatal* class represents a minority (*n* = 33, 8.6%) with symptom levels above the recommended clinical cut-off of 17 at recruitment (mean = 22.1, SD = 4.67), which decline to moderate levels until 3-month postpartum (mean = 12.9, SD = 5.40), but worsen again at 12-month postpartum to reach a mean of 19.3 (SD = 3.49).

Results of the sensitivity analysis, not presented here, suggest that a 2-class model per arm was the most optimal. The trajectories and sample proportions generated for each arm were similar to those presented when both arms were combined.

### Predictors of trajectories

The results of the unadjusted logistic regressions are presented in Table 3. The *antenatal only* class was used as the reference class. None of the baseline demographic variables differed between the two classes. Also, neither employment nor socio-economic status were significant predictors of class, however food insecurity was: the odds of being classified in the *antenatal and postnatal* trajectory were 2.5 times greater (95% CI: 1.21, 5.15; *p* = 0.013) among participants who reported being severely food insecure.

**Table 3:** Unadjusted logistic regression, with *antenatal only* class as reference

	Antenatal & Postnatal (*n* = 33)		
Variable	OR	95% CI	*p* value
Baseline demographic characteristics			
Age	1.05	0.99–1.12	0.112
Education level			
Grade 0–11	ref	–	
Grade 12 or more	0.69	0.32–1.46	0.327
Marital status			
Lives with partner	ref		
Doesn’t live with partner	1.03	0.48–2.19	0.947
Baseline economic measures			
Employment			
Employed	ref		
Unemployed/studying	0.90	0.44–1.84	0.773
Socio-economic status			
Lower wealth	ref		
Higher wealth	0.74	0.36–1.52	0.415
Food status			
Not severely food insecure	ref		
Severely food insecure	2.50	1.21–5.15	0.013
Baseline social characteristics			
Intimate partner violence (IPV)^a^	2.84	1.23–6.52	0.014
Overall social support	0.97	0.95–0.99	0.011
Social support from family	0.91	0.86–0.96	0.001
Social support from friends	0.98	0.93–1.04	0.498
Social support from significant other	0.94	0.88–1.00	0.046
Baseline health characteristics			
Functioning (WHODAS)	1.03	1.02–1.06	0.002
Functioning (FAI score)	5.74	2.82–11.70	< 0.001
Heavy drinking^b^	2.12	1.03–4.37	0.042
HIV positive status^c^	1.52	0.71–3.25	0.278
Current diagnosis of depression (MINI)^d^	2.77	1.32–5.80	0.007
Lifetime diagnosis of depression (MINI)^d^	2.85	1.38–5.87	0.004
High suicide risk (MINI)^d^	2.58	1.19–5.61	0.017

^a^reference is no IPV; ^b^reference is no heavy drinking; ^c^reference is HIV negative status; ^d^reference is absence of diagnosis/risk; *OR* = odds ratio; *95% CI* = 95% confidence interval

Greater overall levels of social support at baseline decreased the odds of belonging to the *antenatal and postnatal* class (OR = 0.97, 95% CI: 0.95, 0.99; *p* = 0.011). However, only a greater level of family support (OR = 0.91, 95% CI: 0.86, 0.96; *p* = 0.001) or greater level of support from a significant other (OR = 0.94, 95% CI: 0.88, 1.00; *p* = 0.046) decreased the odds of being classified in the *antenatal and postnatal* class. Those who reported experiencing IPV at baseline were also 2.8 times more likely (95% CI: 1.23, 6.52; *p* = 0.014) to belong to the *antenatal and postnatal* class.

Besides a HIV-positive status, all other health-related characteristics at baseline were associated with class membership: the odds of belonging to the *antenatal and postnatal* class were greater among participants reporting greater functional impairment, measured with the WHODAS (OR = 1.03, 95% CI: 1.02, 1.06; *p* = 0.002) and FAI (OR = 5.74, 95% CI: 2.82, 11.70; *p* < 0.001); the odds were also greater among participants who reported heavy drinking during pregnancy (OR = 2.12, 95% CI: 1.03, 4.37; *p* = 0.042), had a current (OR = 2.77, 95% CI: 1.32, 5.80; *p* = 0.007) or lifetime diagnosis of depression (OR = 2.85, 95% CI: 1.38, 5.87; *p* = 0.004), and were at high risk of suicide (OR = 2.58, 95% CI: 1.19, 5.61; *p* = 0.017).

## Discussion

The aim of this study was to identify different trajectories of perinatal depressive symptoms and their predictors among low-income South African women at risk of depression antenatally. Through GMM, we were able to identify two subgroups of women with different severity and chronicity of perinatal depressive symptoms: an *antenatal only* trajectory and an *antenatal and postnatal* trajectory. On the one hand, the *antenatal only* trajectory is consistent with previous longitudinal studies reporting a natural remission group [28–31]. This means that, without intervention, and despite initially mild to moderate depressive symptoms during the first or second trimester, the majority of women showed improvements in their symptoms throughout the remainder of the perinatal period. The *antenatal and postnatal* trajectory, on the other hand, suggests that there was a minority of women who did not see their symptoms remit naturally and remained at risk of depression for most of the perinatal period. So, an initial decline in symptom severity throughout pregnancy and first three months postpartum was observed for both trajectories. This suggests that screening within the first weeks after birth to identify women at risk of postnatal depression may not be effective in identifying at-risk women later in the postpartum period.

The findings also suggest that women who are at risk of chronic depressive symptoms can be differentiated from those showing a natural remission on a range of psychosocial and health-related characteristics during pregnancy, other than their initial depressive symptom severity. Women were more likely to belong to the *antenatal and postnatal* class when they reported being severely food insecure, experienced physical or sexual IPV, had lower support from family or significant other and reported problematic drinking during pregnancy. They were also at greater risk of committing suicide and were more likely to have a current or lifetime diagnosis of depression. These findings support previous evidence of the association between perinatal depressive symptoms in South African women and suicide risk [78], hazardous drinking [5, 24], IPV [3, 19, 20] and food insecurity [5, 14, 16, 17]. It is interesting to note that the same association between suicidal risk and trajectories were found when the suicide item was excluded from the HDRS scores used for the GMM and post-hoc analyses (results not presented here). Thus, the association found between suicidal risk and trajectories was not confounded by the inclusion of the suicide item in the HDRS.

Women who were more likely to suffer from chronic depressive symptoms therefore seem to have presented with a higher risk profile during pregnancy in terms of social, economic, health and mental health characteristics. This has important implications on referral and treatment procedures in settings where there are limited mental health resources. First, given the greater likelihood of women diagnosed with depression to be allocated to the ‘antenatal and postnatal’ trajectory, our findings indicate how effective a diagnosis would be in detecting women at higher risk for chronic symptoms. Indeed, some researchers have suggested that conducting a formal diagnostic assessment by a mental health professional could be an efficient use of resources if done in a stepped care manner, that is, if conducted only among women who screened positive on a screening instrument [79, 80]. Alternatively, food insecurity, alcohol use during pregnancy and social support are factors which are relatively easy to assess during pregnancy; only referring to care women with severe symptoms but who also present with greater functional impairment, or who report alcohol use or low support during pregnancy, would limit referrals made and allow the limited mental health services to target women who are at most risk. Women who report severe depressive symptoms but who do not present such risk factors could instead be referred to peer support groups or community-based care.

It is important to note that, despite a decrease in symptoms among the *antenatal only* trajectory, symptom levels remained within the mild range, albeit at the lower end [81], throughout the postpartum period. Yet studies have shown that chronic symptoms, even if mild or moderate, can have adverse effects on child outcomes [27]. It was beyond the scope of this study to assess whether women in different trajectory groups reported different child and maternal outcomes, however future studies should consider investigating the long-term effects of chronic trajectories on mother and child health in LMICs, across all levels of severity.

It is difficult to compare the present findings to those reported by Barthel et al. [33]‘s, the only other study using growth curve mixture models (GCMM) to assess perinatal depressive symptoms among women living in two LMICs. In their study of West African perinatal women, the authors are likely to have excluded a high-risk group by excluding women whose children were born prematurely or had a low birth weight from their study. This is supported by the fact that a chronically severe trajectory was not identified in their study, despite this trajectory being commonly reported in most studies using GCMM [26]. Instead, three trajectories were reported: a chronically low-symptom trajectory, and two transient trajectories, characterised by severe symptoms either early or late in the postpartum period, before returning to low levels [33]. This supports previous studies, which have reported trajectories with initially low levels of depressive symptoms early in pregnancy, which increase later in the perinatal period [30, 82]. It is therefore vital that future studies include women with a range of symptom levels at recruitment, to ensure that all potential trajectories and associated predictors be identified.

### Limitations

The study provides useful evidence on the different trajectories of depressive symptoms of women at risk of depression at their first antenatal visit. However, several limitations need to be highlighted. First, the level of uncertainly associated with the predicted trajectory membership was not controlled for in the post-hoc analyses, which instead treated trajectories as an observed variable. The entropy was above the usual cut-off of 0.80, however, suggesting adequate classification of participants and considered sufficient to conduct post-hoc regression analyses [77]. Second, with a greater sample size, the three-class model identified through GMM may have been more optimal and more representative of the course of symptoms among low-income high-risk women in this setting.

Third, it was established that the reduction in symptoms among the antenatal and postnatal trajectory was unlikely to be due to the psychosocial intervention assessed through the RCT. Indeed, there was no difference in HDRS scores at any point between the control and treatment arms, and the allocation arm was controlled for in the growth mixture model. The sensitivity analysis also revealed that the optimal model generated through GMM was similar when trajectories were generated per allocation arm. However, it is possible that the trajectories identified in this study may partly reflect the combined effect of the enhanced usual care and psychosocial interventions. Indeed, it has previously been suggested that the lack of difference between control and intervention arms, a common phenomenon of RCTs assessing behavioural interventions, may be due to the enhanced usual care having an effect on participants in the control arm, rather than a lack of effect of the intervention itself [83]. Further studies should therefore run the same analyses among women at risk of depression but who did not receive any intervention. A final limitation is the fact that anxiety was not assessed. Anxiety and depression are often comorbid during the perinatal period [13], and the prevalence of anxiety among South African perinatal population is sometimes even greater than that of postnatal depression [4]. Future research should therefore investigate whether comorbid anxiety symptoms predict a different course of perinatal depressive symptoms over the perinatal period.

## Conclusion

This study is one of first studies to investigate the severity and course of depressive symptoms during the perinatal period using GMM in a LMIC. Despite the limitations highlighted above, the findings indicate that perinatal women at risk of depression antenatally cannot be considered as a uniform group. The findings highlight the importance of moving beyond a symptom-based identification of mental illness and towards a screening procedure that takes into account the importance of psychosocial determinants in the development and the course of perinatal depressive symptoms. By doing so, referral systems, as well as timing and target populations for interventions addressing perinatal depressive symptoms can be established in a more efficient way, given the limited mental health resources in low-income South African settings and in other LMICs.

## Additional files

Additional file 1: Results of the preliminary one-class (non-mixture) analyses. Description: Table summarising the statistics generated from one-class analyses – not essential for the main body of the text but may be useful to some readers. (DOCX 12 kb)

Additional file 2: Mean HDRS curves for the alternative 3-class growth mixture model. Description: Figure illustrating the mean HDRS score of participants had they been allocated to three trajectories identified in the alternative 3-class solution of the growth mixture model. (DOCX 69 kb)

## Data Availability

The dataset used and analysed during the current study are not yet made available to the public, but are available from the corresponding author on reasonable request.

## Abbreviations

AIC: Akaike Information Criterion
AUDIT: Alcohol Use Disorder Identification Test
BIC: Bayesian Information Criterion
BLRT: Bootstrap Likelihood Ratio Test
EPDS: Edinburgh Postnatal Depression Scale
FAI: Functional Assessment Instrument
GCMM: Growth curve mixture model
GMM: Growth mixture modelling
HDRS: Hamilton Depression Rating Scale
HFIAS: Household Food Insecurity Access Scale
HICs: High-income countries
IPV: Intimate partner violence
IQR: Interquartile range
LCGA: Latent class growth analysis
LMICs: Low- and middle-income countries
LMRT: Lo-Mendell Rubin Test
MINI: Mini International Neuropsychiatric Interview
MSPSS: Multidimensional Scale of Perceived Social Support
RCT: Randomised controlled trial
WHODAS: WHO Disability Assessment Schedule
