# Supplementary material for: Perinatal depressive symptoms among low-income South African women at risk of depression: trajectories and predictors
Source: BMC Pregnancy Childbirth. 2019 Jun 14;19:202. doi: 10.1186/s12884-019-2355-y (PMC6570971; doi:10.1186/s12884-019-2355-y)
Supplement: Supplementary file 1 — Results of the preliminary one-class (non-mixture) analyses. Description: Table summarising the statistics generated from one-class analyses – not essential for the main body of the text but may be useful to some readers. (DOCX 12 kb) [file 12884_2019_2355_MOESM1_ESM.docx]

Additional file 1. Results of preliminary one-class (non-mixture) analyses

| One-class (non-mixture) models | AIC | BIC | CFI | RMSEA | SRMR |
| --- | --- | --- | --- | --- | --- |
| Intercept only | 8171.806 | 8177.461 | 0.000 | 0.303 | 0.408 |
| Linear | 7962.612 | 8006.069 | 0.079 | 0.265 | 0.170 |
| Quadratic | 7754.626 | 7813.885 | 0.996 | 0.025 | 0.021 |

CFI: Comparative Fit Index; RMSEA: Root Mean Square Error of Approximation; SRMR: Standardised Root Mean Square Residual; NOTE: Can conclude model fits the observed data well when: CFI>0.95, SRMR<0.08 and RMSEA<0.06; smaller AIC or BIC values also suggest better model fit
