# Supplementary figures and images for: Perinatal depressive symptoms among low-income South African women at risk of depression: trajectories and predictors
Source: BMC Pregnancy Childbirth. 2019 Jun 14;19:202. doi: 10.1186/s12884-019-2355-y (PMC6570971; doi:10.1186/s12884-019-2355-y)

Additional file 2. Mean HDRS curves for the alternative 3-class growth mixture model


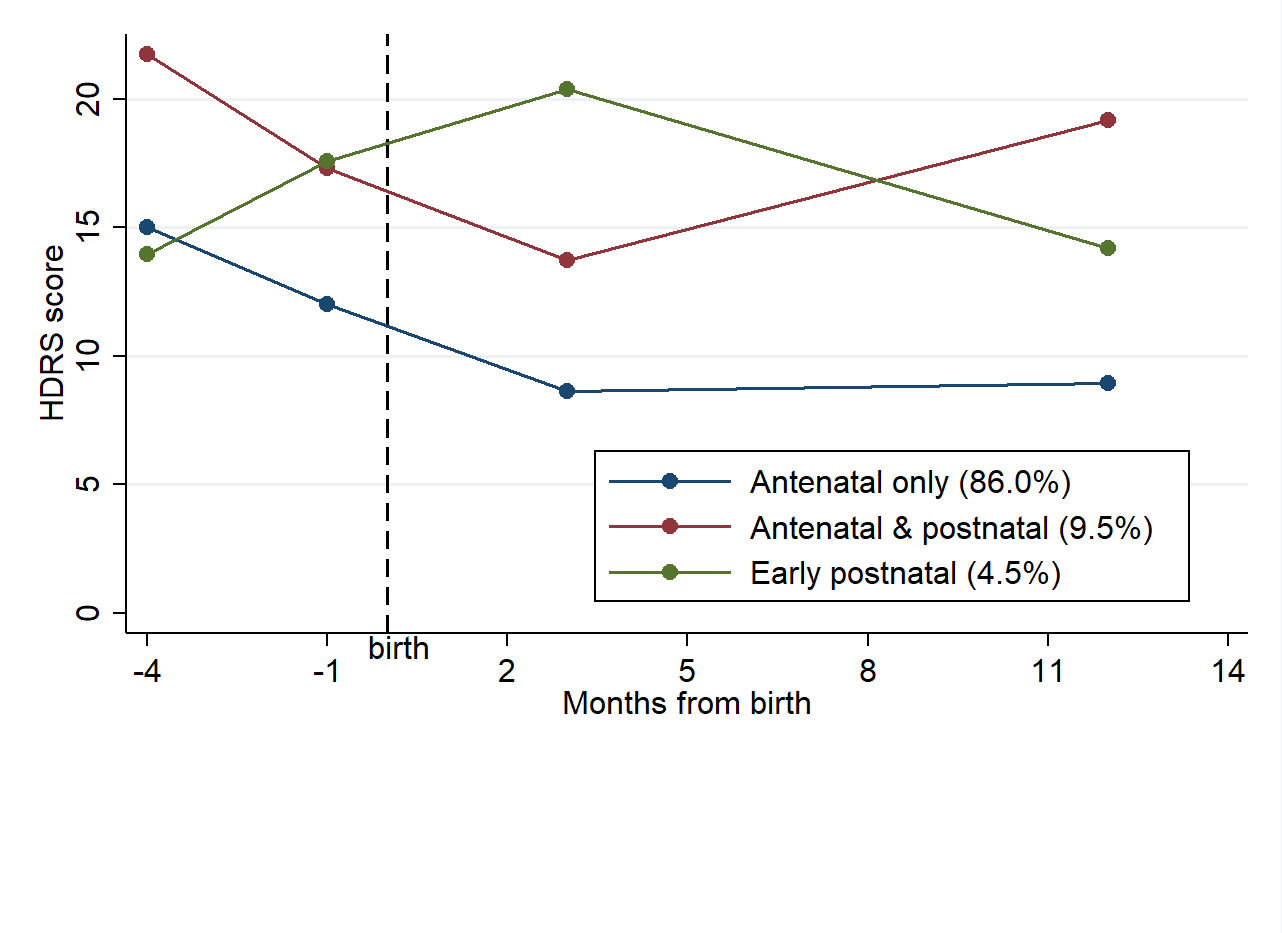

Supplement: Supplementary file 2 — Mean HDRS curves for the alternative 3-class growth mixture model. Description: Figure illustrating the mean HDRS score of participants had they been allocated to three trajectories identified in the alternative 3-class solution of the growth mixture model. (DOCX 69 kb) [file 12884_2019_2355_MOESM2_ESM.docx]
